# Animal Welfare: Focusing on the Future

**DOI:** 10.3390/ani4040779

**Published:** 2014-12-09

**Authors:** Donald M. Broom

**Affiliations:** Centre for Animal Welfare and Anthrozoology, Department of Veterinary Medicine, University of Cambridge, Madingley Road, Cambridge CB3 0ES, UK; E-Mail: dmb16@cam.ac.uk

This book, which is a volume in an OIE series, describes much that is relevant to animal welfare, the chapters being in English with summaries or full text in French and Spanish. As with many reviews of our state of knowledge, many contributions to this volume draw on previous publications. For example, David Fraser’s excellent discussion of the globalization of farm animal welfare is explained at greater length in his 2008 paper [1] and book [2]. However, chapters on drivers of animal welfare policy in Africa, the Americas, the Far East and Australasia and the Middle East are amongst those that are novel. The description by Aidaros of Islamic teachings in relation to animal welfare is particularly welcome.

New developments in thinking about animal welfare, such as Boissy and Lee’s chapter on how to use the links between emotion, motivation and cognition when assessing welfare, are valuable. Also Lawrence and Wall discuss selecting animals for “environmental fit” and hence better welfare and several papers explain how modern technologies can be utilized in order to manage animals better and improve their welfare.

Many of the chapters emphasise the substantial OIE contributions to the subject area in recent years. Welfare is generally presented as the broad scientific concept used by most animal welfare scientists. Nicks and Vandenheede, in their chapter on welfare in relation to health, refer to welfare as involving all aspects of coping with the environment including disease. However, they also accept the rather imprecise 1946 WHO definition of health. In an era before the scientific use of the term “welfare” the WHO defined health as a state of well-being but also incorporated in ‘health’ all of what we now think of as part of welfare. Their definition is contrary to normal public usage in that most people limit health to conditions related to physical or mental pathology. The WHO definition is not easy to use scientifically as both health and welfare can be measured on a scale from very positive to very negative and are not just positive. It is logical to consider health as a part of welfare and not vice versa as do Nicks and Vandenheede.

The welfare of the estimated 400 million draught animals in the world is the subject of two interesting chapters, one by Abdul Rahman and Reed and the other by Tadich and Stuardo focusing on recent work in Chile. The latter reports that most owners of draught equids in their study were poor but responsible and caring. The lack of farriery services in some areas led to poor welfare associated with hoof problems. Other especially useful chapters are those on the welfare of dogs and cats by Sonntag and Overall, non-traditional pets by Schuppli *et al.* and three on fish welfare. Huntingford and Kadri cover farmed fish welfare assessment and problems, Braithwaite and Ebbesson explain pain and stress in fish and Lines and Spence describe humane slaughter of fish. The ethics of laboratory animal usage, humane killing of pest species and killing for disease control purposes are also discussed. In the editors’ conclusions, OIE, FAO, EU, ISO and a range of international veterinary and farm animal association initiatives on animal welfare are mentioned. It is clear that there has been a very substantial increase in national and international activity in this area in recent years and that this will continue.
